# Plastic Surgery of the Breast: Keeping the Nipple Sensitive

**Published:** 2015-07-02

**Authors:** Charles A. Riccio, Matthew R. Zeiderman, Saeed Chowdhry, Ronald M. Brooks, Shahrooz S. Kelishadi, John Paul Tutela, Joshua Choo, David V. Yonick, Bradon J. Wilhelmi

**Affiliations:** Division of Plastic and Reconstructive Surgery, University of Louisville School of Medicine, Louisville, Ky

**Keywords:** nipple innervation, reduction mammaplasty, nipple, breast reconstruction, nipple-areola complex

## Abstract

**Introduction:** Since its inception, reduction mammaplasty has matured considerably. Primary evolution in clinical research and practice has focused on preserving tissue viability. Surgery involves preserving not only tissue viability but also function and sensation. The nipple serves as the sensate unit of the breast and is a valuable part of women's psychological and sexual health, making preservation of nipple sensation of utmost important. Studies regarding primary innervation to the nipple are few and often contradictory. We propose an unsafe zone in which dissection during reduction mammoplasty ought to be avoided to preserve nipple sensation. **Methods:** Circumareolar dissection of 22 cadaveric breasts was performed. Primary nerve branches to the nipple-areola complex were identified and dissected to their origin. **Results:** Three to 5 branches of the fourth intercostal nerve primarily innervated the nipple on 18 of 22 breast dissections. Two breasts received innervation from the third intercostal nerve and 2 from the fifth intercostal nerve. In half of the specimens, accessory innervation from the third and fifth intercostal nerves provided medial branches to the nipple. **Conclusions:** The fourth intercostal nerve provides the major innervation to the nipple-areola complex. Avoiding dissection in inferolateral quadrant “unsafe zone” of the breast during reduction mammaplasty and other breast surgical procedures can reliably spare nipple sensation and maximize patient outcomes.

Since its inception, reduction mammaplasty has matured considerably. Primary evolution in clinical research and practice has focused on developing techniques to preserve tissue viability and breast parenchyma, skin, and nipple tissue. Previously, women with macromastia were more concerned with breast size and shape over mammary sensation. Presumably, the improved aesthetic outcome resulted in an enhanced body image and helped patients feel more sensual. However, surgery today involves preserving not only tissue viability but also function in terms of sensation. The nipple serves as a sensate unit in erectile function and plays a large part in the physical intimacy of women. Nipple sensation has shown to be a valuable part of women's psychological and sexual health. While preservation of nipple sensation is of utmost importance, the literature regarding primary innervation of the nipple is scant and contradictory.^1^^-^5 The authors review the current literature of nipple innervation and perform anatomical studies to identify a safe zone for reduction mammaplasty to preserve nipple sensation.

## METHODS

Eleven dissections were performed on 22 cadaver breasts at the University of Louisville Fresh Tissue Lab. Four cadavers (8 breasts) had macromastia as determined by the investigator's judgment. Circumareolar subcutaneous dissection was performed to identify the nerves from the chest wall to the nipple using 2.5× loupe magnification. Once the trajectory of the nerves to the nipple was identified, the nerves were dissected back to their origin of penetration of the chest fascia.

## RESULTS

**Figure 1:**
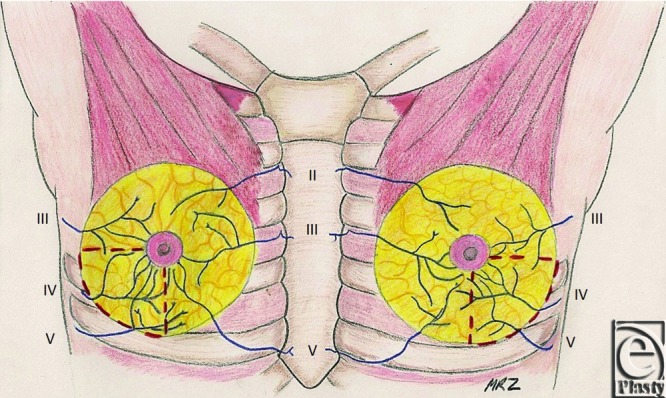
Anterior view of intercostal nerve innervation to the nipple. The red dashed lines demarcate the inferolateral breast quadrant to be avoided during surgical dissection so as to preserve nipple sensation.

**Figure 2:**
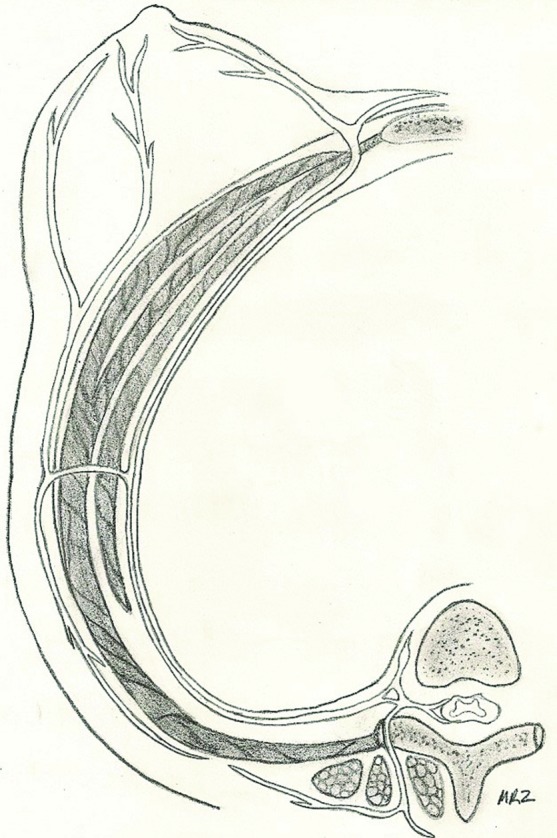
Cross-sectional illustration of intercostal nerve innervation to the nipple.

**Figure 3:**
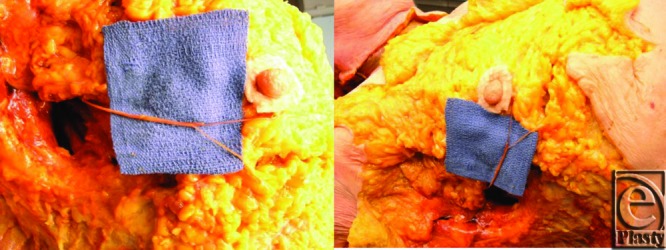
Photographs from cadaveric dissection, highlighting the course of the fourth intercostal nerve in the inferolateral quadrant.

Anatomical results identified 3 to 5 branches of the fourth intercostal nerve to primarily innervate the nipple on 18 of 22 breast dissections. Two breasts received innervation from the third intercostal nerve and 2 from the fifth intercostal nerve. In half of the specimens, accessory innervation from the third and fifth intercostal nerves provided medial branches to the nipple (Table 1 and Fig 1). On the left side, the nerve travels toward the nipple at the 4 o'clock position while it enters at the 8 o'clock position on the right side. The nerve pierces the chest fascia above the fifth rib 3 cm lateral to the border of the pectoralis major muscle and travels through the gland in an inferolateral position toward the nipple (Figs 2 and 3). Breast size did not alter the course of the intercostal nerves to the nipple-areola complex (NAC).

## DISCUSSION

Breast-reduction surgery has evolved considerably through the centuries. Prior to the late 1800s, breast amputation was the procedure performed to eliminate excessively large breasts. Theodore Galliard-Thomas was the first to advocate preservation of some part of the glandular tissue in the 1880s.^6^ The mid-1920s brought the techniques of Lexar and Kraske to transpose the nipple after creating subcutaneous flaps.^6^ Thorek^7^ was the first to perform a free nipple graft for excessive macromastia. Schwarzman et al^8^ in the 1960s developed the concept of de-epithelialization to maintain the nipple complex on a dermal plexus. Wise^9^ built upon Biesenberger's procedure of separating the skin from the gland and transposing the nipple by developing resection patterns to aid in safer more reliable reductions.^10^ The vertical bipedicle dermal reduction was popularized later by McKissock.^11^ Inferior pedicle techniques were developed by Robbins^12^ and Courtiss and Goldwyn.^13^ Courtiss^14^ later described using liposuction alone as a reduction method. The vertical reduction was later popularized by Arie, Lassus, Lejour, and Hall-Findlay.^15^^-^21 Primary goals of these procedures through the years have been tissue viability, shape, contour, and scar aesthetics.

However, many advocate that nipple sensation is paramount to patient satisfaction as well. As the nipple is perhaps the most sensitive area of the breast, it serves a significant role in a woman's sexual life. Erectile function and sensation are frequently necessary for both the woman herself and her partner. Consequently, loss of these functions has a detrimental impact on procedure outcome and patient satisfaction.^22^^-^26

Previous studies have demonstrated that the majority of women feel that nipple-areola sensitivity as an important part of their sexual life, and of those women who underwent breast surgery and lost nipple sensation, the majority of women were significantly bothered by the result.^26^

In general, patients undergoing breast-reduction surgery demonstrate high satisfaction due to the improvement in neck, shoulder, and back pain. However, loss of sensation to the nipple results in a poorer outcome. Anatomical analysis of the innervation of the NAC possibly helps guide the surgeon in avoiding damage to the nerves of the nipple. Our anatomical study demonstrated the innervation of the nipple to come laterally from 3 to 5 branches off of the fourth intercostal nerve. In addition, in some specimens, intercostal nerves 3 and 5 provided accessory innervation. These findings were consistent in both normal and hypertrophied breast specimens. Breast size did not alter the trajectory of the nerve to the NAC. Our results demonstrate that the distortion of breast tissue observed in obese patients and patients with macromastia does not alter the anatomical course of innervation to the NAC. Furthermore, the stretching of breast tissue observed with aging as a result of loss of support by the suspensory ligaments was not observed to alter anatomical course of the intercostal nerves to the nipple. The fourth intercostal nerve pierces the fascia of the fifth rib just lateral to the border of the pectoralis major muscle. The nerve travels to the NAC through the inferolateral position of NAC. Previous studies have demonstrated the lateral branch of fourth intercostal nerve to be the most reliable innervation to the NAC.^1^^-^5 Other studies also demonstrated accessory innervation of the nipple to come from both anterior and lateral branches of the second through sixth intercostal nerves. 1^,^^3^ However, not all innervation to the NAC can be reliably salvaged during reduction mammoplasty.

Lessons learned in the anatomy laboratory demonstrate that the plastic surgeon ought to avoid excessive resection and dissection in the inferolateral areas of the breast so as to preserve the innervation of the NAC. Breast size does not appear to alter the course of the intercostal nerves through the breast parenchyma. Consequently, we propose that the findings of this anatomical study can be extrapolated for guidance of breast surgery in patients with either normal or hypertrophied breast tissue. Avoidance of the inferolateral quadrant “unsafe zone” during reduction mammoplasty and other breast surgical procedures can prevent damage to the fourth intercostal nerve and accessory innervation by the third and fifth intercostal nerves. Such technique will reliably maintain the primary innervation of the nipple and maximize patient satisfaction.

Frequently, the plastic surgeon must individualize therapy to the patient. A fixed procedure does not always apply to every clinical scenario. Adhering to principles of techniques and knowledge of anatomy frequently serves as a foundation for the reconstructive surgeon when planning procedures. This study can aid the novice and experienced surgeons in obtaining quality outcomes in terms of not only aesthetics but also function.

## CONCLUSION

Preserving nipple sensation is a valuable goal in breast surgery. Many women value nipple sensation as a significant component of sexuality and quality of life. The innervation of the nipple is predictable based on anatomical findings. An unsafe zone can reliably be avoided in the inferolateral area of the breast. Clinical application of these findings demonstrates the possibility to reliably maintain the nipple as an aesthetic and sensate unit.

## Figures and Tables

**Table 1:** Primary and accessory innervation of the nipple*

Specimen	Side	ICN	Accessory ICN	No. of branches
1	L	4	3	3
2	L	4	5	3
3	R	4	5	5
4	R	5	5	5
5	L	4	3	4
6	R	4	3	4
7^†^	R	3	4	4
8^†^	L	4	5	3
9	L	4	5	5
10	R	4	3	3
11^†^	L	4	3	4
12^†^	R	4	3	3
13	L	3	5	5
14	R	4	5	5
15	L	4	3	4
16	R	4	3	5
17	R	5	4	3
18^†^	L	4	5	5
19^†^	R	4	4	4
20	L	4	3	5
21^†^	L	4	3	4
22^†^	R	4	5	3

*Specimen data for 22 dissections equally distributed between the left and right sides. Eighteen of 22 dissections showed primary innervation from the fourth ICN. Accessory innervation came from ICN 3 to 5. The primary nerves have 3 to 5 branches to supply the nipple.

†Indicates macromastia.

ICN indicates intercostal nerve; L, left; R, right.

## References

[1] Schlenz I, Kuzbari R, Gruber H, Holle J (2000). The sensitivity of the nipple-areola complex: an anatomic study. Plast Reconstr Surg.

[2] Sarhadi NS, Shaw-Dunn J, Soutar DS (1997). Nerve supply of the breast with special reference to the nipple and areola: Sir Astley Cooper revisited. Clin Anat.

[3] Sarhadi NS, Shaw Dunn J, Lee FD, Soutar DS (1996). An anatomical study of the nerve supply of the breast, including the nipple and areola. Br J Plast Surg.

[4] Jaspars JJ, Posma AN, van Immerseel AA, Gittenberger-de Groot AC (1997). The cutaneous innervation of the female breast and nipple-areola complex: implications for surgery. Br J Plast Surg.

[5] Craig RD, Sykes PA (1970). Nipple sensitivity following reduction mammaplasty. Br J Plast Surg.

[6] Purohit S (2008). Reduction mammoplasty. Indian J Plast Surg.

[7] Thorek M (1989). Possibilities in the reconstruction of the human form 1922. Aesthetic Plast Surg.

[8] Schwarzman E, Goldan S, Wilflingseder P (1977). The classic reprint. Die Technik der Mammaplastik [The technique of mammaplasty]. Plast Reconstr Surg.

[9] Wise RJ (1956). A preliminary report on a method of planning the mammaplasty. Plast Reconstr Surg (1946).

[10] Harris HI (1953). Advantages of the Biesenberger technic for mammaplasty of hypertrophied pendulous breasts. J Int Coll Surg.

[11] McKissock PK (1972). Reduction mammaplasty with a vertical dermal flap. Plast Reconstr Surg.

[12] Robbins TH (1977). A reduction mammaplasty with the areola-nipple based on an inferior dermal pedicle. Plast Reconstr Surg.

[13] Courtiss EH, Goldwyn RM (1977). Reduction mammaplasty by the inferior pedicle technique. An alternative to free nipple and areola grafting for severe macromastia or extreme ptosis. Plast Reconstr Surg.

[14] Courtiss EH (1993). Reduction mammaplasty by suction alone. Plast Reconstr Surg.

[15] Arie G (1957). Una nueva tecnica de mastoplastia. Rev Iber Latino Am CirPlast.

[16] Lassus C (1986). An “all-season” mammoplasty. Aesthetic Plast Surg.

[17] Lassus C (1987). Breast reduction: evolution of a technique—a single vertical scar. Aesthetic Plast Surg.

[18] Lejour M, Abboud M, Declety A, Kertesz P (1990). [Reduction of mammaplasty scars: from a short inframammary scar to a vertical scar]. Ann Chir Plast Esthet.

[19] Lassus C (1970). A technique for breast reduction. Int Surg.

[20] Hall-Findlay EJ (1999). A simplified vertical reduction mammaplasty: shortening the learning curve. Plast Reconstr Surg.

[21] Hofmann AK, Wuestner-Hofmann MC, Bassetto F, Scarpa C, Mazzoleni F (2007). Breast reduction: modified “Lejour technique” in 500 large breasts. Plast Reconstr Surg.

[22] Terzis JK, Vincent MP, Wilkins LM, Rutledge K, Deane LM (1987). Breast sensibility: a neurophysiological appraisal in the normal breast. Ann Plast Surg.

[23] Slezak S, Dellon AL (1993). Quantitation of sensibility in gigantomastia and alteration following reduction mammaplasty. Plast Reconstr Surg.

[24] Gonzalez F, Brown FE, Gold ME, Walton RL, Shafer B (1993). Preoperative and postoperative nipple-areola sensibility in patients undergoing reduction mammaplasty. Plast Reconstr Surg.

[25] Temple CL, Hurst LN (1999). Reduction mammaplasty improves breast sensibility. Plast Reconstr Surg.

[26] Schlenz I, Rigel S, Schemper M, Kuzbari R (2005). Alteration of nipple and areola sensitivity by reduction mammaplasty: a prospective comparison of five techniques. Plast Reconstr Surg.

